# ‘It’s my home and your work’: the views of a filmed vignette describing a challenging everyday situation from the perspective of people with intellectual disabilities

**DOI:** 10.1080/17482631.2018.1468198

**Published:** 2018-05-07

**Authors:** Ove Hellzen, Marit Haugenes, May Østby

**Affiliations:** a Centre of Care Research, Steinkjer, Norway; b Department of Nursing Sciences, Mid-Sweden University, Sundsvall, Sweden; c Faculty of Health Sciences, Nord University, Namsos, Norway; d Faculty of Health Sciences and Social Care, Molde University College, Molde, Norway

**Keywords:** Content analysis, ethical issues, focus-group interviews, intellectual disabilities, vignettes

## Abstract

**Purpose: **Examining everyday challenges in the interactions between people with intellectual disabilities and their staff, as seen from the user’s perspective, is an important perspective in health care research. Involving people with intellectual disabilities as so-called co-researchers is a relatively unexplored research strategy. In this paper, co-researchers participated in all the steps of the research process, from planning to reporting, in addition to the written reporting of the findings. The aim of this study was to explore how people with intellectual disabilities experienced a filmed vignette of an everyday situation. **Method: **Based on audio-recorded and transcribed individual and focus-group interviews with people with intellectual disabilities, performed by co-researchers with intellectual disabilities together with researchers, qualitative content analysis was used. **Results:** The analysis reveals three themes: “being emotionally touched”, “being aware of the other”, and “being unclear”. **Conclusions:** The results are discussed in light of normalization and participation in society with independence and one’s own decision-making. Regarding the care of people with intellectual disabilities, the main finding is the need to focus not only on greater involvement of this population in their own daily lives, but also to teach self-determination skills. Another finding is the importance of involving people with intellectual impairment as co-researchers.

## Introduction

This paper is part of a larger study designed to examine everyday challenges in the interactions between people with intellectual disabilities and their staff, as seen from both their perspectives. In this paper, which is the first paper, we present findings from the point of view of people with intellectual disabilities. Identified challenges were exemplified in six short (approximately 2–4 minutes) filmed vignettes showing interactions between staff and users (all filmed vignettes available in Norwegian at http://naku.no/node/1341). The constructed vignettes are based on problematic everyday situations involving people with intellectual disabilities when their own homes become another person’s workplace. Narrations were collected by people with intellectual disabilities participating in the study as co-researchers. Involving people with intellectual disabilities as so-called co-researchers is a relatively unexplored research strategy (Inglis & Cook, 2012). In the definition of co-researcher, the emphasis is on the prefix “co”. People with intellectual disabilities are not independent researchers in the traditional sense. The condition is that traditional researchers initiate the research and support the co-researchers in their work. In the study, co-researchers participated in all the steps of the research process, from planning to reporting the findings.

The constructed vignettes were transferred into six filmed vignettes, cast and directed by members of a local theatre group in central Norway. During autumn 2012, the vignettes were recorded by a local professional film team. Researchers and co-researchers were divided into three research teams from each county that constituted the research area for the study. The three research teams conducted qualitative interviews and focus-group interviews with people with intellectual disabilities, staff and disability nursing students.

Traditionally, disability studies have been dominated by the social sciences, where research, particularly in the Nordic countries, has focused primarily on disability policies and the daily lives of disabled people (e.g., Tøssebro, 2013). In the literature, the need for support when public services are provided in private homes is highlighted and documented (Harris, Beringer, & Fletcher, 2016). However, the majority of research focusing on the homes of people with disabilities has been linked to living conditions and, to a lesser extent, the content of the services; that is, about how support workers negotiate the complexity of risk management and the promotion of autonomy in their daily practice (Björnsdóttir, Stefánsdóttir, & Stefánsdóttir, 2015).

Historically, people with intellectual disabilities have lacked access to individual autonomy. They have not been allowed to make their own choices, based on the perspective view that, due to their impairments, they were not capable of doing so (Carlson, 2010). In order to achieve diversity in society, people with disabilities need to receive help and support to make their own decisions. In the literature, supported decision-making is seen in different areas; for example, in the areas of intellectual disability (Hoole & Morgan, 2011), end-of-life care (Ekdahl, Andersson, & Friedrichsen, 2010), and mental health (Mahone et al., 2011). The central principle underlying supported decision-making is autonomy (Chartres & Brayley, 2010). For instance, in a Nordic context, disability is viewed in relational terms and understood as the result of the discrepancy between the disabled person’s capabilities and the functional demands made by the society. Therefore, people are defined as disabled if they face barriers in everyday life due to limited abilities, disease or other impairments (Tøssebro, 2004).

According to Wehmeyer, Avery, Mithaug, and Stancliff (2003), self-determined behaviour refers to actions that can be identified by four essential characteristics—the person acts autonomously, the behaviours are self-regulated, the person initiates and responds to events in a psychologically empowered manner, and the person acts in a self-realizing manner. However, a conflict may be seen in the daily relationship between people with intellectual disabilities and staff; this conflict can be viewed as one between independence in private life and the responsibility of minimizing risks, and it risks contrasts to ideas about citizenship and the equality of all people (Gustavsson, Tøssebro, & Traustadóttir, 2005). In the context of intellectual disabilities, reducing one’s self-determination is seen as infringing on the individual’s personal dignity. In the literature, dignity is defined as, on the one hand, a universal or absolute quality that all human beings have to the same extent as long as they live (Nordenfelt, 2004). In everyday reality, there are relational challenges arising in the relationship itself that are influenced by the staff’s ability to reflect and act. On the other hand, the people receiving the care have their history and patterns of response that are expressed in the meeting. Therefore, supporting adults with intellectual disabilities may be understood as managing two potentially conflicting duties: a duty of care, which requires support workers to protect service users from potential harm, and a duty to recognize and promote service users’ autonomy (Hawkins, Redley, & Holland, 2011).

Recognizing autonomy requires allowing individuals to take risks. According to Meininger (2001) among others, respect for personal autonomy is a central value in public policy documents, which also include the recognition of autonomy for people with intellectual disabilities. In everyday life, this means that person-centred planning and acting should be the basis of communal service delivery. This means that the focus is put on the individual staff member’s competence and ethical skills and how these affect the user’s possibility of influence and self-determination. Traditionally, the literature on autonomy and people with intellectual disabilities has focused on perspectives of parents and professionals (e.g., Carter et al., 2013). The voices of people with intellectual disabilities have, with few exceptions (e.g., McKelvey, Morgaine, & Thomson, 2014), been absent from this discussion.

Traditionally, the care of people with intellectual disabilities is based on the assumption of what Vatne (2009) describes as complementarity care; that is, that the care provider provides services based on what are perceived as the care recipient’s needs and that the recipient receives the assistance offered. According to Stiker (1999), people with disabilities are expected to imitate people with non-disabilities and strive for normality. Limitation as professional caring activity is something that is often taken for granted, which may be due to the fact that care activities can often be related to everyday activities and governed by social norms. Limitation is a commonly used practice in caring for people with intellectual disabilities that risks being experienced by the receiver as abusive and degrading, a method that may create barriers to autonomy and empowerment.

Autonomy and empowerment in relation to people with intellectual disabilities is an important issue. It is connected to international human rights treaties, national legislation and policy (e.g., United Nations, 2006). Despite this, people with intellectual disabilities have lacked a voice, authority, and control over their lives throughout history. Only recently have people with intellectual disabilities been acknowledged as valuable contributors to the discussion concerning intellectual disabilities (Wahmsley & Johnson, 2003). Therefore, staff involved in daily activities play an important role in the users’ well-being, as they have influence in the residents’ daily decision-making. There is always a risk that individual staff reflect and act based on their own projections and experiences in the meeting with the individual user (Dunn, Clare, & Holland, 2010), a fact that gives primacy to personal values and life experiences (Dunn, Clare, & Holland, 2008). Therefore, the following question arises: How do users with intellectual disabilities experience questions of autonomy and empowerment in practice? To increase our knowledge, the aim of this study was to explore how people with intellectual disabilities experienced a filmed vignette of an everyday situation.

## Method

This is a qualitative and descriptive study based on both a focus-group discussion and individual interviews with people with intellectual disabilities. Qualitative content analysis was used to illuminate participants’ reflections about one filmed vignette describing a difficult everyday situation focusing on the interaction between one woman with intellectual disabilities and a staff member.

### Research group and co-researchers

Information about the co-researchers and their participation in the research process is more meticulously reported in a forthcoming paper. In this paper, we provide only a short overview of the research group, including the co-researchers, and its work structure. Three people with intellectual disabilities from three counties in Mid-Norway were consecutively recruited to participate in the research as co-researchers. Together with three researchers from three different university colleges in the middle of Norway and one researcher from the Centre of Care Research, Mid-Norway, the co-researchers formed a research project group.

**Figure 1.**
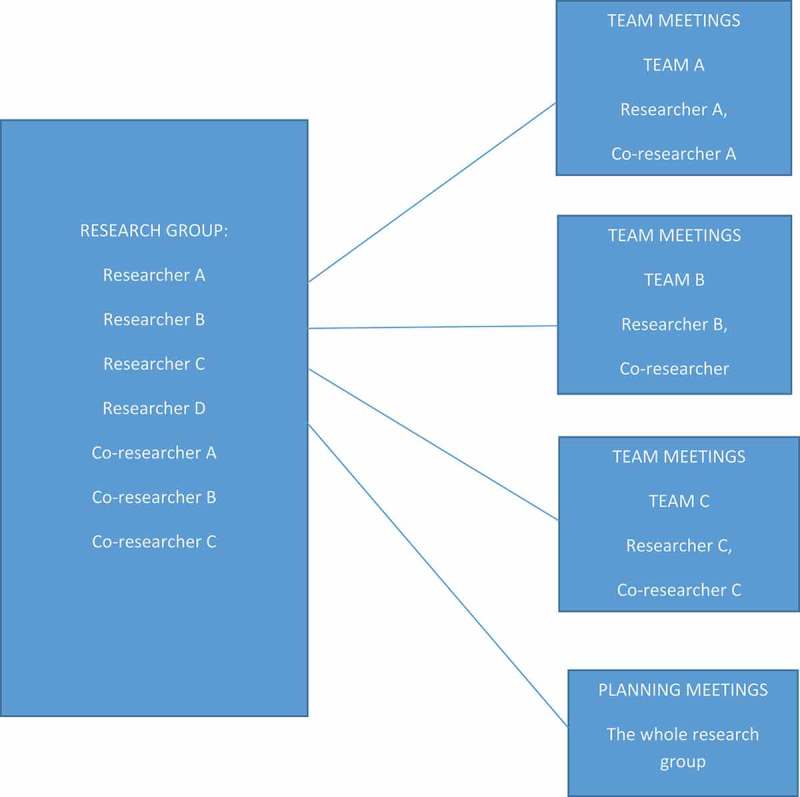
Work structure of the project.

Together with the researchers, the co-researchers formed three research teams as dyads. Each research team was located in the same county where the individual co-researcher lived. The purpose of including co-researchers in the study was that they have personal lived experiences of receiving help in their own homes and therefore were best suited to formulate specific problem areas related to receiving help in one’s home; from these, vignettes were constructed. This means that the project group was a composite group, comprising both people without and with intellectual disabilities, which showed that the latter’s inclusion and participation are possible in research projects. Through regular project meetings and planning meetings every other month with the whole research project group, and team meetings once a week within the individual research teams, the project group succeeded in their work despite large distances between them. The reasons for those meetings were to exchange ideas, opinions, research-based findings, and analyses, and to develop vignettes. An overview of the work structure of the project is shown in Figure 1.

**Figure 2.**
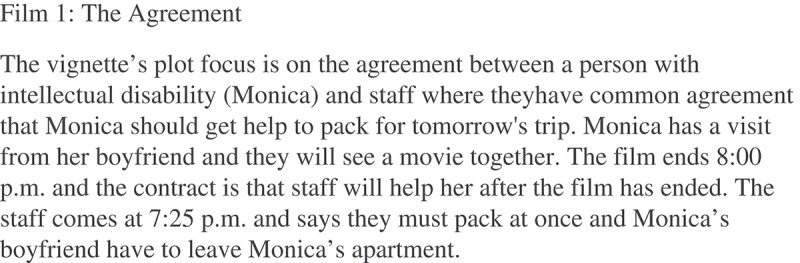
Vignette presented to the users (Available in Norwegian an as a text version in English: http://naku.no/node/1341).

### Participants

An informational letter was sent to the unit managers asking them to inform residents about the aim of the study. Users who met the inclusion criteria and were willing to participate in this study could provide their names to the responsible person in the municipality. The inclusion criteria were being a person with an intellectual disability, having the ability to verbally communicate, having experience of receiving professional help at home, and having consent competences. Staff were informed of the interview performance and were asked to thoroughly inform patients about what the interview meant and how it would be conducted. Those who expressed interest in the study to the unit manager were contacted to determine a time and place suitable for the interview. At the time of the interview, the interviewers (researcher and co-researcher) verbally informed participants about the performance of the interview. In total, eight people with intellectual disabilities (four men; four women), aged 25–55 years old, from two different municipalities in the middle of Norway consented to participate. However, a male participant took advantage of the opportunity to leave the study without explaining why. One focus-group discussion including three female participants and individual interviews with three male participants were conducted. At the time of the interviews, one participant was considered not to have consent competence and therefore was omitted from the interview. In total, six participants were included in the study.

### Data collection

This study combines qualitative data from focus-group discussions and individual interviews (Lambert & Loiselle, 2008). The interaction among the focus-group members provided broad and rich data (Peek & Fothergill, 2009), and the individual interviews provided a deeper understanding of the users’ lived experiences (Roper & Shapira, 2000).

One audio-recorded focus-group discussion with three people with intellectual disabilities and three individual interviews were conducted between January and March 2013. One of the research teams was present at the focus-group discussions, with each member of the dyad alternating between being a moderator and being an observer. Individual interviews were conducted by one of the other research teams. Both focus-group discussions and individual interviews took place in a conference room without interruptions or disturbances at the specific municipality. A thematic interview guide related to the aim of the study was used. First, the co-researcher verbally informed the interviewees about the study and its aims, and informed them that they were free to withdraw their participation at any time during the interview if they chose to.

A filmed episode was presented that showed a typical interaction between a staff member and a user (see Figure 1), based on the experiences offered by a consulting board consisting of people with intellectual disabilities. The vignette presented to the users is shown in Figure 2.

The initial query was “Can you please tell us what you think about when you see this video?” Then, participants were asked to reflect on the vignette based on the following five questions:

- Can you please tell us what you think about the staff member in this episode?
- Can you please tell us what you would have done in this situation?
- Please tell us why you think the staff member acts the way she does.
- Can you please tell us what you think would have happened if Monica had refused to pack her suitcase before eight o’clock?
- Is there anything more you want to say about the film?

During the focus-group discussions, follow-up and clarifying questions were asked and comments made, such as “What would you feel/think/do in this situation?” and “Tell me more about that.” The focus-group discussions lasted between 25 and 35 minutes. The individual interviews were conducted parallel to the focus-group discussions with similar follow-up questions. This offered the opportunity to compare participants’ answers and see if there were any differences between reflections made alone and those made in a group and whether any deeper understanding of the users’ lived experiences of similar situations as those seen in the film sequence could be gained. The individual interviews lasted between 47 and 81 minutes. All data were audio-recorded and transcribed verbatim by the researchers.

### Qualitative content analysis

The interviews were analysed using a qualitative content analysis. Content analysis is a systematic research method used to describe a specific phenomenon at manifest and latent levels (Graneheim & Lundman, 2004). Between what is expressed in the text and its interpreted meaning (Downe-Wamboldt, 1992; Kondracki, Wellman, & Amundson, 2002). Content analysis can be described as a process of linking the underlying meaning from categories into themes on an interpretive level (Baxter, 1994; Woods & Catanzaro, 1988). Before the analysis began, the authors transcribed the interviews verbatim. The analysis was performed in several steps. First, we listened to the tapes together and performed several readings of the texts to gain a sense of the whole. Second, each interview was read several times to obtain a sense of the content. Third, based on the aim of this study, the text was divided into meaning units—words, sentences, or whole paragraphs—that were related to each other by their context and content. The meaning units were condensed, while still preserving their meanings, and were labelled with codes. The codes were abstracted and sorted into four categories, which were interpreted to form three themes. Due to the intertwined nature of the participants’ experiences, the themes were not mutually exclusive (Graneheim & Lundman, 2004). The first author of this manuscript performed the data analysis. All authors contributed to the discussion to confirm the findings.

### Ethical considerations

The Norwegian Social Science Data Services (No. 31,120) granted permission for the research. The study was performed according to the ethical guidelines described in the Declaration of Helsinki (World Medical Association, 2013). The participants received verbal and easy-to-read written information about the aim of the study, the voluntary nature of participation, their right to withdraw without specifying why, and the confidential nature of the study. There was also a risk that the participants might feel violated by our close questioning. However, there was no interdependence between the researchers and the interviewees, and they could leave the study whenever they wished.

## Findings

The analysis of the interviews resulted in three themes with four categories (Table I). The themes and categories are presented in the text below and illustrated by quotations from the interviews.

**Table I.** Overview and examples from content analysis.

Meaning unit	Condensation	Code	Category	Themes
I have thought about it a bit, but no, that I can’t do it	Have thought about it, I can’t do it	Lack of self- respect	To appear to be obedient	Being unclear
I think she was a bit disappointed, I think. I think maybe she’s hurt inside '…' I think	She was a bit disappointed, hurt inside	Feeling hurt	To be able to see the others, to read others’ feelings	Being emotionally touched

### Theme 1: being emotionally touched

This theme was characterized by the experience of being emotionally touched. Participants were affected by broken agreements and the emotions that these elicited. They recognized the emotions experienced by Monica (the name of the user in the episode).

#### To be able to see the others, to read the feelings of others

All participants had difficulties expressing their thoughts in direct connection to the vignette. For example, when asked what they thought about the film, one of them said: “No, it’s not so much, I do not have much to say, but …', while another said, Nice apartment '…' gets help then. However, in regard to the direct question of putting themselves in Monica’s situation, How does Monica feel?, some of the participants expressed that she has feelings of sadness, disappointment, and embarrassment, and that, on the whole, she does not feel comfortable in the situation. One of them said: 'I think she was a bit disappointed, I think. I think maybe she’s hurt inside … I think.' They all said they had often experienced similar situations themselves. One of the participants said: I have experienced it many times … I have lived at different places. When I lived in [name of the place], where I lived for 13 years, during which I was not allowed to do what I wanted'. All participants had had experiences of broken agreements, and, according to one participant, every user has experiences of these soon or later.

#### To feel frustration over a broken agreement

Most interviewees provided descriptions of their opinions about signed agreements and what they meant. They all agreed that an agreement should not be broken. One of them said: “No, she had a deal … Then it was said that there would be eight o’clock. Then she came [at] half past seven instead … when they have a deal … so they have a deal.” ……According to one of the participants, Monica had a just way out of the situation—to pack. One said: 'packing must come first'. They all decided that she would have had a problem if she had spoken up and refused to do as the staff member said. Several of the participants said that the situation encountered was difficult. Saying no to staff is not easy. One of them said: 'No, I have not much to say.' All of them saw the difficulty of coping with the situation in the filmed vignette. One said: 'Must come later … it’s somewhat difficult … come a little later.'

### Theme 2: being aware of the other

This theme revealed one category: being aware of the other. This can be described as the participants’ defence of the staff member’s behaviour, even if they did not like it.

#### Views on staff

All participants had difficulties expressing negative opinions about the staff member in the vignette. They described seeing the staff member as competent and as doing nothing wrong, even if she did break an agreement. They said that the staff member “was a little difficult … not exactly difficult but she was so busy”. A common view is that staff are always in too much of a hurry. Instead, they should be calmer. One participant said: ‘No, she could have taken it a bit easier.’ This determined an attitude among participants that they did not like to meet in staff general terms, and they said that ‘they shall not be so, at least I think so’.

“However, some of them explicitly expressed complaints about staff in the interviews, but always in general terms: ‘when a staff [member] visits a person’s home, he shouldn’t be angry’. According to one person, ‘if I say no, it will become trouble … this isn’t easy”’. Even if participants admitted during interviews that they did not like all staff, they noted that they have to do as the staff wish. One said: “I’m kind. I say yes; I don’t say no.”

### Theme 3: being unclear

This theme revealed the second category: being unclear. This can be described as participants trying to mask their feelings.

#### To appear to be obedient

Participants described that they act against staff by being accommodative. This approach helps them maintain a sense of safety. Participants experienced it as hard to say no to the staff. One said: “I have thought about it a bit, but no, I can’t do it.” Another participant expressed it this way: ‘I’m so then, that I can’t say … I’m good to say yes to everything. I cannot say no.’ To be friendly, even if they want to say no, is common. In that way, participants could postpone a possible conflict. One said: “No I don’t do that. I’m just always saying yes.”

## Discussion

An important step towards normalization and full participation in society with confirmed rights is being given increased opportunities for independence and the right to make one’s own decisions. However, even if one belongs, in many respects, to a vulnerable group, there must be a balance between giving an individual responsibility and independence and, at the same time, providing an adequate amount of help and support to a person with an intellectual disability. Our study shows that participants’ management of a broken agreement was characterized by negative emotions that were masked by maintaining a positive exterior and the participants’ positive view of the staff member and her conduct. The imbalance between the level of individual participant choice and staff control is influenced by the uniquely created environment that exists in the care of people with intellectual disabilities (e.g., Kåhlin, Kjellberg, & Hagberg, 2016). This imbalance was described in three themes: *being emotionally touched*, *being aware of the other*, and *being unclear*. All themes included experiences that might be seen as violating the dignity of the person with intellectual disabilities. Nordenfelt (2004) was of the opinion that dignity, on the one hand, is a universal or absolute quality that all human beings have to the same extent as long as they live. On the other hand, dignity is formed by the culture and society and depends on a person’s self-image. This kind of dignity can be lost due to having an intellectual disability that affects a person’s competence and status as an independent person in the community.

This study showed that people with intellectual disabilities might be emotionally affected by a broken agreement. *Being emotionally touched* in this study could be understood as being exposed to an emotive situation where the person opened himself or herself to the other’s vulnerability. In this study, participants felt sympathy towards the woman in the vignette and were emotionally involved through the physical milieu and specific situation. They have personal experiences of similar milieus and situations. Participants may relate to the vignette and the situation in a distanced, reflective way or in an emotionally engaged, immediate way. In the latter instance, the understanding of the experience, just as the feelings that it provokes, seems to be both spontaneous and inseparable. According to Lyons and Sullivan (1998), well-functioning relationships with other people are central to self-identity and self-respect and can signify the difference between isolation and social integration (Paterson & Stewart, 2002).

The inability that people with intellectual disabilities experience means that they are not able to exert any influence over the events of their own daily lives, which may create apprehension and apathy. Self-decision means a person’s ability to make choices and take control of his or her life (Burke, 2005), which contributes to positive outcomes for an individual with intellectual disabilities (Wehmeyer & Gragoudas, 2004) and empowers the individual to speak for himself or herself (Rapaport, Manthorpe, Moriarty, Hussein, & Collins, 2005). In this study, instead of making their own decisions, the participants took the side of the staff member and defended her actions even if it was at the expense of their own autonomy.

Logically, if people suffering from advanced-stage dementia are able to make decisions, people with intellectual disabilities are able to do it, too. In this study, participants were *being aware of the other* when they defended the staff’s actions. This attitude is supported by previous studies showing that people with intellectual disabilities act in a defensive way and at the expense of their own decision-making (Goodley, 2000; Olney, 2001). In light of the self-efficacy theory, such institutional constraints can negatively influence a person’s self-efficacy beliefs and well-being (Bandura, 1997).

We interpret this undemanding environment at community-based housing in municipal apartments as being a barrier to one’s own decision-making. It seems that, in this theme, one may find the crux of the tensions and power hierarchies that contribute to people living with intellectual disabilities not being able to say no and thereby lacking empowerment and the ability to say “This is not what we agreed.” However, poor experiences of empowerment and the ability to make decisions about one’s life may reinforce negative feelings regarding an indivdual’s identity, seen as low self-esteem, lack of self-worth, and poor self-identity. In light of the self-efficacy theory, such institutional constraints can negatively influence a person’s self-efficacy beliefs and well-being (Bandura, 1997). Therefore, it is important to break down barriers and to provide individuals with intellectual disabilities with opportunities to speak up and make choices, to have a voice, and to find new pathways (Caldwell, Arnold, & Rizzolo, 2012).

The theme *being unclear* refers to people with intellectual disabilities attempting to be accommodative towards staff. According to Olney (2001), staff often assume that people with severe challenges such as intellectual disabilities are so globally impaired that they cannot know what they want. Furthermore, staff seem to have a selective view of their interactions with people with intellectual disabilities; that is, certain communications are rewarded with attention, while others are ignored. Brown, Gothelf, Guess, & Lehr, (1998) stressed that, rather than command and enforce obedience in their interactions with people with intellectual disabilities, staff should make it their goal in the interaction to expand their understanding of the desires of the users and, through that, increase opportunities for real choice for people with intellectual disabilities. In this study, it becomes obvious that the participants wish to say no, but that they are, over the years, “disciplined” to say “yes and to be obedient and kind”. This is probably the key in regard to how staff exert power and use their positions, possibly without even realizing that they are doing this. Instead, people with intellectual disabilities could be empowered, and professionals could view them as competent communication partners and respect their competence and autonomy (e.g., Brown et al., 1998).

In the overall project, the main aim is to help people with intellectual disabilities who receive help at home to speak up and reveal how they want their lives to be. By verbalizing disagreement with the staff and speaking up about the things that are important for them to decide for themselves, decision-making and autonomy may increase for people with intellectual disabilities. Although this research is exploratory, it puts the focus on the relationship between autonomy and communication difficulties. It appears that having difficulties with communication may have a negative influence on an individual’s autonomy and decision-making. It seems logical to assume that participants’ disabilities or needs for support influence their capacity for self-determination and decision-making in daily life (Wehmeyer & Garner, 2003). Therefore, an important aspect in the care of people with intellectual disabilities is not only to aim at greater involvement in their own daily lives, but also to teach self-determination skills (Arndt, Konrad, & Test, 2006).

### Methodological considerations

When using a vignette in the research context, it is important to keep in mind that being responsive and having a sense of understanding of other people’s subjective situations affects one’s relationship to oneself; that is, an understanding of oneself in relation to other people (Gadamer, 1994). This means that, when viewing a vignette focusing on meeting people in vulnerable situations, the participants meet not only the actors in the vignette; they also meet themselves (Chen, Del Ben, Fortson, & Lewis, 2006).

People with intellectual disabilities can be involved in research if they have the right conditions and are supported by traditional researchers. Accepting co-researchers’ capacity and empowering their participation as co-researchers in the research process has been the key to success in this study. Support required for a co-researcher goes beyond practical support; it involves developing a relationship that can actively challenge views and foster reflection. In this project, people with intellectual disabilities have been involved in all steps of the research project where the focus has been on research about ethical, demanding everyday situations for people receiving professional help in their own home.
